# Fur-rubbing with *Piper* leaves in the San Martín titi monkey, *Callicebus oenanthe*

**DOI:** 10.5194/pb-4-127-2017

**Published:** 2017-06-26

**Authors:** Rosario Huashuayo-Llamocca, Eckhard W. Heymann

**Affiliations:** 1 Proyecto Mono Tocón, Jr. Reyes Guerra No. 430, Moyobamba, Peru; 2 Facultad de Ciencias Biológicas, Universidad Nacional San Luis Gonzaga de Ica, Av. Los Maestros s/n, Ica, Peru; 3 Verhaltensökologie und Soziobiologie, Deutsches Primatenzentrum, Leibniz-Institut für Primatenforschung, Kellnerweg 4, 37077 Göttingen, Germany

## Abstract

We report observations on fur-rubbing with leaves from *Piper aduncum* by a San Martín titi monkey, *Callicebus oenanthe*. Fur-rubbing occurred during the transition from the dry to the rainy season in a titi monkey group living in a forest fragment in the Moyobamba region of Peru. Since *Piper *leaves include very potent compounds that may affect ectoparasites, we tentatively interpret the observed fur-rubbing as self-medication.

## 1 Introduction

Several primate and at least one carnivore species rub their fur with arthropods or their secretions, plant material (leaves, resin) or other substances, including man-made substances (tobacco smoke, soap), both in the wild and in captivity (Birkinshaw, 1999; Bowler et al., 2015; Gasco et al., 2016; Gompper and Hoylman, 1993; Huffman, 1997, 2011; Morrogh-Bernard, 2009; Nolte, 1958). Repelling or killing ectoparasites and microbial pathogens is invoked as the principal function of fur-rubbing (Baker, 1996; Falótico et al., 2007; Valderrama et al., 2000; Weldon et al., 2003), but it may also be involved in social bonding and olfactory communication (Campbell, 2000; Leca et al., 2007; Paukner and Suomi, 2012).

For titi monkeys, Carrillo-Bilbao et al. (2005) report fur-rubbing with chewed leaves of *Tetrathylacium *in *Callicebus discolor* (*Plecturocebus discolor*) from Yasuní, Ecuador, and with chewed leaves of five Annonaceae species and one Bignonaceae species in *Callicebus toppini* (*Plecturocebus toppini*) from Manu, Peru. In this paper, we report the use of *Piper aduncum* leaves by the San Martín titi monkey *Callicebus oenanthe* (*Plecturocebus oenanthe*), observed during an ecological study on this endemic and highly endangered species.

## 2 Methods

### 2.1 Study area

**Figure 1:**
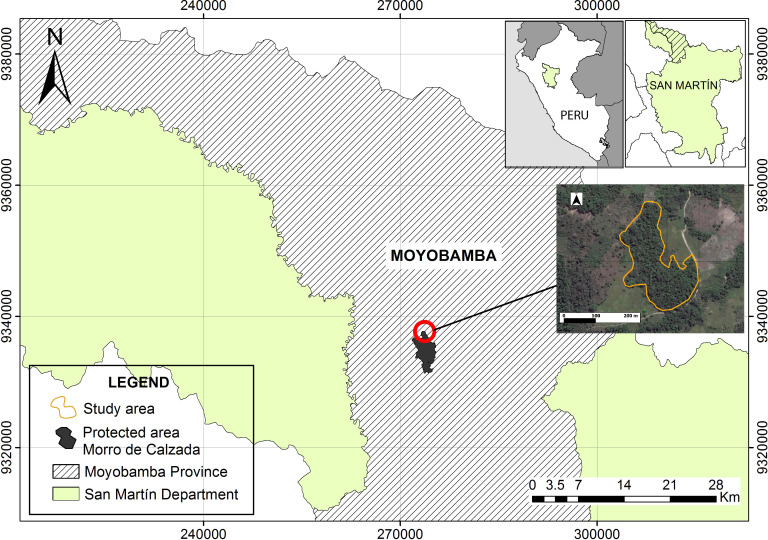
Location of the study area.

**Figure 2:**
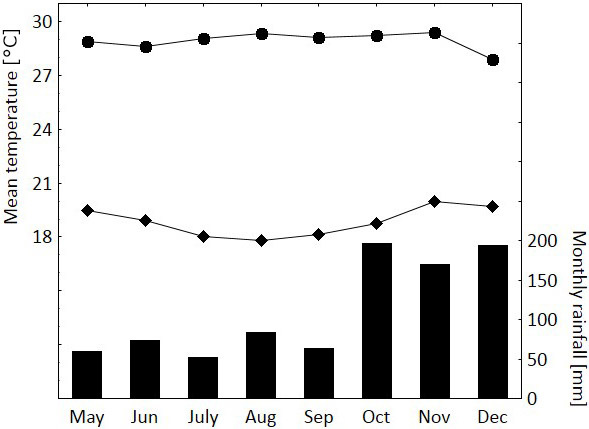
Mean daily maximum temperature (•), mean daily minimum temperature (⧫) and total monthly rainfall (bars) during the study period May–December 2014. Data from http://www.senamhi.gob.pe/main_mapa.php?t=dHi (accessed 5 April 2017).

The observations were made in a 4 ha forest fragment surrounded by pastures and agricultural fields in the district of Yantaló, province of Moyobamba, department of San Martín (UTM coordinates: 9338221.4 northing, 274918.8 easting, 18M; Fig. 1). The fragment is located at the periphery of the protected area “Morro de Calzada”. The vegetation is dominated by species of Vochysiaceae, Moraceae, Lauraceae and Bignonaceae. It reaches 12–15 m height and includes a dense understory (Romero Herrada, unpublished data). Average annual temperature is 22.8 ${}^{\circ }$C and annual precipitation around 1350 mm with < 100 mm per month from May to August (climate-data.org, 2017). During our study period, September also received < 100 mm rain (Fig. 2).

### 2.2 Study group and observational methods

The study group included one adult male, one adult female, one subadult female and one juvenile female. It was observed between May and December 2014, for a total of 238 h. We used instantaneous scan sampling for determining the activity budget and use of space, focal animal sampling for feeding behaviour and behaviour sampling (for rare but significant behaviours like the one reported here).

## 3 Results

During focal animal observations, the adult male was seen three times in September and October biting off a leaf from spiked pepper trees, *Piper aduncum*, and squeezing it with both hands. Then the squeezed leaf was rubbed against the abdominal area. This behaviour lasted between 5 and 15 min and involved either a single leaf or up to four leaves.

The adult female was also observed rubbing the abdominal area, but we never saw the collection of *P. aduncum* leaves. We could not see whether the female may have used material smaller than *P. aduncum* leaves for fur-rubbing. Fur-rubbing was not observed in the subadult and juvenile females.

## 4 Discussion

*Piper aduncum*, known in Peru as “matico”, is widely distributed in the Neotropics. It is used in traditional medicine for a broad spectrum of ailments. This includes both internal and external (topical) use of leaf extracts, infusions and decoctions (Taylor, 2006). Constituents of *P. aduncum*, like dillapiole and other phenylpropanoids, are potent insecticides (Fazolin et al., 2014; Marques and Kaplan, 2015; Pino et al., 2011; Piton et al., 2014; Ribeiro et al., 2016; Vila et al., 2005; Volpe et al., 2015). Extracts of *P. aduncum* have also been shown to possess antifungal properties (Santos et al., 2013). While it is unclear whether extracts obtained by squeezing the leaves render all these bioactive constituents, it is highly likely that at least some constituents are released. Therefore, it is plausible to assume that the fur-rubbing with *P. aduncum* leaves observed in *C. oenanthe* functions as a topical self-medication, as suggested for several other primate species. Wild capuchin monkeys, *Cebus capucinus*, and captive owl monkeys, *Aotus* spp., have been reported to use leaves from *Piper marginatum* for fur-rubbing (Baker, 1996; Zito et al., 2003).

Fur-rubbing was observed in September and October, which are months that mark the fast transition from the relatively dry to the wet season (Fig. 2). We speculate that it is related to repelling or killing ectoparasites like ticks, chiggers or mosquitoes that might increase with the onset of the rainy season. For instance, the tick *Amblyomma longirostre* that infests a large number of birds, and terrestrial and arboreal mammals (mainly rodents) throughout the Neotropics, is known from the Moyobamba region (Nava et al., 2010). *Alouatta guariba* and *Sapajus nigritus* are infested by different species of *Amblyomma* (Guglielmone et al., 1990; Martins et al., 2015), and larval *Amblyomma* were the most common ectoparasite of *Leontopithecus rosalia* (Wilson et al., 1989). As ticks have several life stages (larvae, nymphs, adults), their response to climatic conditions can be quite complex, but some species may show higher activity of adults during the rainy season (Labruna et al., 2002; Lacerra de Souza et al., 2006). In sifakas, *Propithecus edwardsi*, ectoparasite richness was higher in the wet compared to the dry season in all habitats, as was the intensity of tick and fly infestations in disturbed habitats (Wright et al., 2009). Mouse lemurs, *Microcebus griseorufus*, were infested by ticks during the dry season, and males have higher infestation rates than females due to more frequent use of the ground (Rodriguez et al., 2015). Infestation by chiggers also varies seasonally (e.g. Dietsch, 2005), and mosquitoes are more abundant during the rainy season.

Although our observations are anecdotal and information on ectoparasites in the Moyobamba region is scanty, our report expands the number of primate species that exhibit topical application of leaves with medical potential.

## Data Availability

There are no further data apart from the observations reported in the paper.
